# Women’s economic independence and physical intimate partner violence (IPV) during separation

**DOI:** 10.1371/journal.pone.0326529

**Published:** 2025-06-20

**Authors:** Stefania Molina, Lena Wagner, Michaela Kreyenfeld

**Affiliations:** 1 Hertie School, Berlin, Germany; 2 Humboldt-Universität zu Berlin, Berlin, Germany; 3 Einstein Center Population Diversity, Berlin, Germany; Clarkson University, UNITED STATES OF AMERICA

## Abstract

A growing number of studies have addressed the issue of intimate partner violence (IPV). While some studies have shown that women’s economic independence reduces the risk of physical IPV, the empirical evidence is still inconclusive. In particular, little is known about the factors that are associated with the likelihood of experiencing physical IPV during the immediate separation process. We use data from the German Family Panel (pairfam) for the years 2009–2022. The analytical sample consists of women who went through a separation since the last interview (n = 786 person-years). We use logistic regression models to examine the determinants of physical IPV during separation. We find that mothers with minor children have significantly higher odds of experiencing physical IPV during separation than women who do not have minor children (OR=1.96, p=.006), controlling for key sociodemographic characteristics. Low education as well as non-employment are associated with higher risks of physical IPV during separation for both women with and without minor children. However, non-employment is a particular risk factor for mothers with lower levels of education. Divorce and separation, especially when minor children are involved, can be a highly stressful time when partner conflict escalates and, in some cases, culminates in violence against the partner. This paper highlights the importance of maternal labor market integration and economic independence as a lever to potentially reduce physical IPV and conflict during separation.

## Introduction

Intimate partner violence (IPV) encompasses a range of behaviors within intimate relationships that profoundly impact the well-being of victims/survivors [1]. It manifests in forms such as physical aggression, sexual coercion, psychological abuse, and controlling behaviors by current or former intimate partners [2]. While affecting individuals of all genders, empirical evidence highlights women’s disproportionate representation among victims/survivors [3]. Previous research underscores its status as the most common form of violence against women and a significant public health issue [1,4], emphasizing the disparate health outcomes experienced by female victims/survivors [5]. The consequences of IPV extend beyond immediate physical and psychological trauma, permeating all aspects of victims’ lives and reverberating throughout society [6].

Several risk factors associated with IPV have been identified, including low education, low income, and having several young children in the household [7–10]. However, the relationship between women’s economic independence and IPV is ambiguous, reflecting the complex interplay between power dynamics within couples and women’s varying abilities to end a partnership before conflict escalates [11–12]. From a bargaining theory perspective, economic resources strengthen a woman’s bargaining position within the household [13,14]. A woman who contributes financially is less economically dependent on her partner, which may enable her to negotiate more favorable dynamics within the relationship or to leave an abusive partner altogether. In line with gendered power theories, women’s economic independence may challenge traditional norms of male dominance within the household and potentially reduce a male partner’s ability to exert coercive control [15]. Furthermore, if a woman works, the household likely relies on more resources than a single breadwinner household would. As higher household income reduces the risk of poverty and financial strain, it may also eliminate one of the main sources of conflict and stress in a partnership.

Empirical evidence on the relationship between markers of women’s economic independence and experience of IPV is mixed. Some studies show that women’s education, labor market integration, and higher earnings reduce IPV [10–12], while other studies report that women’s labor market participation may be associated with elevated risk of IPV [16,17], particularly when the male partner is out of work [7,17]. Such findings support the claim that in some couple constellations, women’s empowerment may threaten the male partner’s gender identity, resulting in a “male backlash” [16]. However, evidence for such a “male backlash” often comes only from cross-sectional research [18–20]. In many cases, women only separate after experiencing prolonged IPV, making economic independence less about prevention and more about enabling them to leave a violent partner. As such, woman’s economic independence serves as a protective factor against IPV, as it strengthens her ability to leave a relationship before dysfunctional conflict starts or escalates. During this period, women may expand their labor market engagement in the anticipation of the termination of the union, but cross-sectional data are unable to uncover the complex relationship between partnership conflict, women’s labor market participation, and the termination of a partnership.

Victimized women often seek to end a physically abusive relationship through separation and divorce. However, during the separation process, the level of conflict frequently intensifies [21], and may be accompanied by verbal and physical aggression [22]. Being economically dependent on her partner may inhibit a woman from leaving a dysfunctional relationship early, before the conflict level escalates [23]. Therefore, it may be assumed that women’s economic independence lowers the risk of a conflictual and physically abusive divorce and separation. While several studies have examined whether separated women have an elevated risk of IPV [24,25], few have isolated the separation process as a distinct period of heightened risk or explored how pre-separation economic conditions influence violence during this specific transition. Most existing studies focus on IPV either during the relationship or after separation, without closely examining the dynamics and risk factors present during the separation process itself. This paper seeks to fill this research gap. By drawing on data that include information on physical violence during the separation process, we focus on a well-defined event in the life course. Moreover, we use panel data, which allows us to measure women’s employment status before separation, and reduces the risk of bias arising from women experiencing physical IPV ending a relationship and entering employment as a result of physical IPV or separation.

Although our study can shed light on the relationship between women’s economic independence and physical IPV during the separation process, there are several aspects that it does not examine. First, the study focuses on physical IPV and does not address other forms of IPV. While most quantitative studies on IPV focus on physical IPV [11], other forms of violence, such as emotional violence or coercive control and financial abuse, can occur either independently or alongside physical violence. We cannot address these other facets of IPV, as the data we are using does not include them. Second, as we focus on IPV during separation, we disregard the fact that many women may not be able to leave a relationship, despite being subjected to abuse. Qualitative studies have identified many factors contributing to the inability to leave, such as physical entrapment, social isolation, learned helplessness, economic dependence, concerns about child custody, unwarranted optimism about the abuser’s behavior, and strong reservations about divorce or separation, often influenced by religious and moral beliefs [26–29]. This means that we cannot generalize our findings to all women. Our results are limited to separated women and provide insights into the relationship between physical IPV during the separation process and women’s economic independence. Third, we draw on longitudinal data and are thus able to measure women’s employment status in the year prior to separation. However, it is possible that women experiencing abuse during separation were also abused earlier in the relationship. Women in abusive or unstable relationships might seek employment as a form of economic security or independence, anticipating that they will later separate. As a result, our estimates may still be downward biased, as we measure employment only in the year preceding separation, and not necessarily in the year when the woman formed the intention to separate.

## Methods

### Data

This study uses data from the German Family Panel (pairfam), a longitudinal dataset launched in 2008 to investigate family dynamics in Germany [30]. The survey follows a cohort design, and includes respondents from the 1971–73, 1981–83, and 1991–93 birth cohorts (the 2001–03 cohort was added in wave 11). It should also be noted that East Germans are oversampled in the data, which we account for by controlling for region of residence in the regressions. The German Family Panel is an annual survey that had roughly 14,000 respondents in the first wave. We select waves 2–14 (2009–2022), as information on our outcome of interest was not collected in the first wave. The pairfam data can be considered high quality, as the panel stability is consistently above 80% from wave 3 onwards, and the attrition rates are between 6% and 14% [31].

The sample is restricted to women who separated from their married or cohabiting partner in the year preceding the survey. Note that if children are present, the separation may be from the father of the children, but it may also be from a stepparent. Furthermore, separation is not an absorbing event, so several separations may be recorded per woman. We account for this in the regression by using clustered standard errors. The final sample consists of 714 separated women who contribute 786 person-years to the investigation. In other words, since some women were observed for more than one year and each year of observation is counted separately, the total number of observations contributed by all women adds up to 786.

### Measures

The outcome variable identifies women who experienced physical intimate partner violence during the separation process. Women who were partnered at the last interview but had since separated were asked the following question: *“In the past year up to the point of your separation, were there any arguments between you and your ex-partner during which either of you used physical force?”* The binary outcome variable takes the value of 1 if the woman answered *“Yes, due to my partner”*; or *“Yes, due to my partner and me equally”.* The variable takes the value of 0 if the women said *“No”.* We exclude women who answered *“Yes, due to me”* (less than 5%) as, in these cases, the violence is not directed against the women. Approximately 17% of women reported experiencing physical violence during separation. In the majority of these cases (74%), the violence came only from the male partner. In 26% of the cases, the violence came from both partners.

We distinguish between women with a post-secondary or higher degree (high education level) and women with less education (low education level). We also differentiate between women who work and women who do not work (due to unemployment, educational participation, or other reasons). While we measure education at the time of the interview, we measure employment in the year before the interview, and therefore before the separation. Education and employment are crucial for women seeking to achieve economic independence. Education provides women with the skills and knowledge they need to gain access to employment opportunities and high wages, which, in turn, contribute to their financial autonomy.

The control variables are migration background (no migration background vs. migration background), age at interview (18–24 vs. 25–34, 35–50), and region (East vs. West Germany). Younger and migrant women have been associated with a higher risk of IPV [7,10]. Whether women are categorized as East or West German is based on their place of residence at the time of the survey: those living in the eastern German states (including Berlin) are categorized as East Germans and those living in the western German states are classified as West Germans. We control for East and West Germany because even though more than 30 years have passed since German reunification, the demographic patterns in the two formerly separate states still differ strongly. These differences pertain to childbearing behavior [32], but also to maternal employment, gender roles, and the availability of public daycare [33]. It is easier for East German women than for West German women to achieve economic independence due to the wider coverage of daycare services and the more liberal gender role attitudes in the Eastern states. We also distinguish between women who do and do not have minor children (under age 18). Partners’ characteristics (such as his education, employment, or alcohol consumption) would be a relevant determinant of IPV. However, we were unable to incorporate them due to a high number of missing values in the partner-related data.

Table 1 displays the summary statistics of the analytical sample by whether women have minor children. Among other things, the table shows that among the sample of women with minor children, a larger fraction lives in East Germany. The sample reflects the differences in the demographic behavior in the two parts of the country, and the fact that childlessness is still lower in the Eastern than in the Western states [34]. Note that the share of East Germans in the sample is generally elevated, as East Germans are oversampled in pairfam data. About 16% of the women in the sample have a migration background. The share of women who are not employed, less educated, or older are overrepresented in the group of women with minor children.

**Table 1:** Sample statistics, column percent.

	Without minor children	With minor children	Total
No migration background	83.66	83.77	83.72
Migration background	16.34	16.23	16.28
West Germany	74.87	58.17	66.28
East Germany	25.13	41.83	33.72
Age 18–24	32.28	6.68	19.11
Age 25–34	51.44	41.34	46.24
Age 35–50	16.27	51.98	34.65
Employed in year before separation	76.70	65.10	70.74
Not employed in year before separation	23.30	34.90	29.26
Less than post-secondary education	54.93	65.35	59.80
Post-secondary education or higher	46.07	34.65	40.20
No physical IPV during separation	86.13	80.45	82.21
Physical IPV during separation	13.87	19.55	16.79
Person-years	382	404	786

*Note.* Data are from pairfam, waves 2–14, unweighted own estimates

### Analytical strategy

The analysis uses logistic regression models where the outcome variable equals 1 if the separation was characterized by physical IPV during separation, and 0 otherwise. We first estimate a model that includes all women (Model 1) and then conduct a separate analysis for women with and without minor children (Model 2a and 2b). In an interaction model, we examine whether employment moderates the effect of education, and whether the patterns differ for women with and without minor children (Model 3a and 3b).

## Results

Table 2 shows the logistic regression model results for the binary outcome variable ‘physical IPV during separation’ (Model 1). The analysis shows a highly elevated risk of physical IPV for women with a migrant background (OR=1.97, *p* = .010). Compared to East Germans, West German women also have an elevated risk of physical IPV, but the parameter is not statistically significant at conventional levels. Older women (beyond age 35) face a lower risk of physical IPV than younger women. The main variables that indicate vulnerability and economic dependence are whether women have minor children, have lower education, and are not employed. All of these factors are strongly related to physical IPV during separation. Having minor children increases the risk by 96% (*p* = .006). One possible explanation for this elevated risk is that the intensive care duties associated with raising small children may increase stress and tension within the relationship, exacerbating conflict. Moreover, a mother might be trapped in an abusive relationship because she fears losing custody or not being able to financially support her children. Additionally, a mother caring for young children may be less likely to be in full-time employment due to her caregiving responsibilities. These limitations on a woman’s economic independence may reduce her ability to leave an abusive partner, thereby prolonging her exposure to physical IPV. The model also shows that the odds of experiencing physical IPV during separation among those who obtained having less than post-secondary education is 149% higher (*p* < .001) compared to the reference category (post-secondary education or higher), while the odds among non-employed respondents is 72% higher (*p* = .010) compared to being employed. Therefore, our model strongly supports the hypothesis that factors that facilitate women’s financial autonomy contribute to lower physical IPV levels during separation.

**Table 2:** Results from the logistic regression model with physical IPV during separation as the dependent variable. Odds ratios and 95%-CI.

	Odds ratio	95% CI	*p*
No migration background	Ref.		
Migration background	1.97	1.17**—**3.29	.010
West Germany	Ref.		
East Germany	0.80	0.50**—**1.29	.361
Age 18–24	Ref.		
Age 25–34	0.65	0.38**—**1.13	.124
Age 35–50	0.28	0.14**—**0.55	<.001
No minor children	Ref.		
Minor children	1.96	1.21**—**3.16	.006
Less than post-secondary education	2.49	1.54**—**4.01	<.001
Post-secondary education or higher	Ref.		
Employed in year before separation	Ref.		
Not employed in year before separation	1.72	1.14**—**2.60	.010
Constant	0.10	0.06**—**0.19	<.001
N	786		

*Note.* Data are from pairfam, waves 2–14, unweighted own estimates. Clustered standard errors.

**Fig 1:**
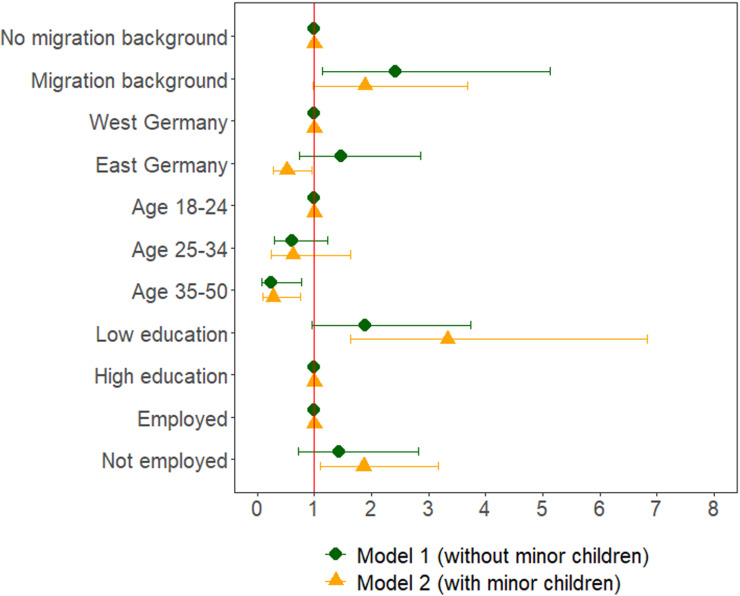
Results from the logistic regression model with physical IPV during separation as the dependent variable. Odds ratios and 95%-CI. *Note.* pairfam, waves 2-14, unweighted own estimates. Clustered standard errors. Low education includes “less than post-secondary education” and high education includes “post-secondary education or higher.” Employment is measured in the year before separation.

Fig 1 displays the results from the separate models for women without minor children (Model 2a) and women with minor children (Model 2b). Overall, the patterns are very similar for the two groups, albeit with some notable differences. First, the region matters only for women with minor children. For West German mothers, the risk of physical IPV during separation is strongly elevated (OR=1.89, p = .039) compared to that for East German mothers. As was noted earlier, gender role patterns, maternal employment rates, and the availability of public daycare differ greatly between East and West Germany [33]. More conservative gender roles and less conducive conditions for combining work and family life might make separation in West Germany more conflict-ridden, resulting in a higher incidence of physical IPV during separation if minor children are involved in West than in East Germany. The model results also show that the effect of having low education on the risk of physical IPV is more pronounced for women with minor children than for other women. Finally, for women without minor children integration into the labor market does not greatly affect the odds of experiencing physical IPV, whereas for women with minor children the risk of physical IPV is strongly elevated if they do not pursue paid employment (OR=1.84, p = .024).

**Fig 2:**
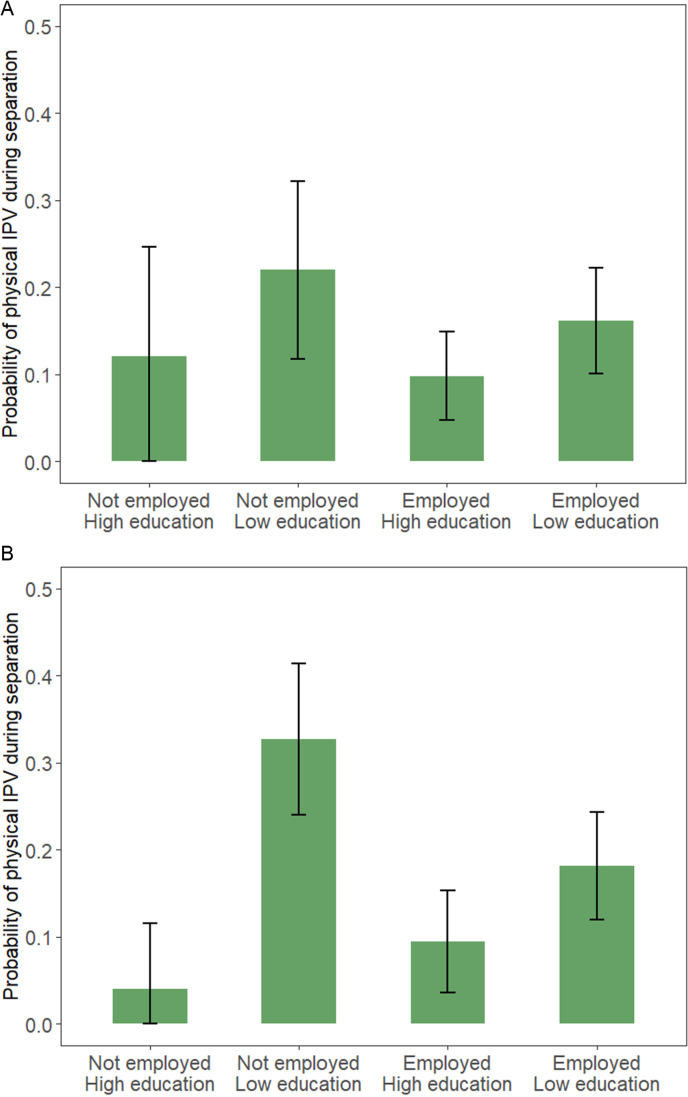
Average predicted probabilities and 95%-CI from the logistic regression model with physical IPV during separation as the dependent variable. **a.** Women without minor children (Model 3a). **b.** Women with minor children (Model 3b). *Note.* pairfam, waves 2-14, unweighted own estimates. Clustered standard errors. Low education includes “less than post-secondary education” and high education includes “post-secondary education or higher.” Employment is measured in the year before separation.

The final part of the investigation includes the interaction model in which separate investigations are conducted for women with and without minor children and employment status is interacted with the level of education. The results are presented in Fig 2 (Models 3a and 3b). To facilitate visualization and interpretation of the interaction effects, we calculate the average predicted values from the models. While the analysis for women without minor children (Model 3a) shows some variation across groups, no statistically significant differences between groups are found. By contrast, in the analysis for women with minor children (Model 3b), a high-risk group stands out: Mothers with low education who are not working have a much higher risk of experiencing physical IPV during separation (estimated probability of physical IPV = 32%, 95%-CI = 24%−41%) than the other groups. Highly educated mothers face a low risk of physical intimate partner violence during separation, regardless of their employment status. Conversely, the predicted probability of low educated mothers experiencing physical IPV is 33% when they are not employed, compared to 18% when they are employed. The difference between these groups is statistically significant (OR for low educated and not employed = 2.27; *p* = .009, with the reference category being low educated and employed).

## Discussion

This study has examined women’s odds of experiencing physical IPV during separation. Data came from waves 2–14 (2009–2022) of the German Family Panel, which asked respondents who had separated since the last survey year whether they had experienced physical violence in the year leading up to the separation. We found that 17% of women reported subjection to physical violence in the year leading up to their latest separation. Among these, 74% reported that physical violence came only from their male partner, while 26% reported mutual physical violence, meaning that both partners engaged in physical violence. Our operationalization of physical IPV includes cases where physical violence either came only from the male partner or where both the partner and the woman exercised physical violence. We chose this approach because even in instances of mutual physical violence, women reported being subject to physical IPV by their partner during separation. However, we acknowledge that this definition may conflate different dynamics of physical IPV, including potentially reactive or defensive forms of violence exerted by women. Including both cases in the outcome variable provides a broader picture of physical IPV during separation but may blur distinctions between unidirectional and bidirectional violence.

It is essential to recognize that the separation process is often accompanied or preceded by various forms of IPV, including emotional abuse, economic control, and psychological distress. This means that a large fraction of the women in our sample may have been exposed to physical IPV not only during separation but also prior to it. A recent study conducted for Germany highlights that among surveyed women, 15% report having ever experienced physical IPV [35]. A government-mandated study, which was based on a representative sample of 10,000 women aged 16–85, reported a 23% lifetime prevalence [36]. These results align with levels of physical IPV found in this present analysis but it must be borne in mind that they are not fully comparable, as our research focuses on physical IPV during separation, whereas these studies looked at lifetime prevalence for all women, regardless of partnership history.

A main finding from our investigation is that women’s economic independence was associated with a lower risk of physical IPV during the separation process, as the odds of experiencing physical IPV were significantly lower for women with higher levels of education and for women in employment. A separate analysis by motherhood status uncovered stark East-West differences, with West German mothers facing a higher risk of physical IPV during separation than East German mothers. The East-West differences may be related to the enduring disparities in gender roles and women’s levels of economic autonomy in the two regions [33]. Although major efforts have been made since 2005 to expand childcare for children under age three, childcare availability – and thus the ability of mothers to combine work and family life – is less developed in the West than in the East. Compared to their West German counterparts, East German women are more likely to work full-time after having a child, and their greater financial autonomy may put them in a better position to leave a dysfunctional relationship before conflict escalates.

Our analysis also identified a group at particularly high risk. Women with minor children who had less than post-secondary education and who were not working had higher odds of experiencing physical IPV during separation. Of the women in this group, around one-third reported experiencing physical IPV during separation. Prior studies have indicated that unemployment, low income, and debt are significant determinants of separation, and these factors often contribute to increased conflict within families [37]. Less educated women frequently face challenges such as unemployment, financial instability, and high stress levels, which can, in turn, exacerbate conflict and increase the risk of physical IPV during this vulnerable period. Our findings align with prior research suggesting that economic dependence can limit women’s ability to leave abusive relationships early, thereby increasing their exposure to physical violence (e.g., [10]). Moreover, the demands of caring for young children can heighten stress and tension in a relationship, potentially intensifying conflicts [38]. Theoretically, these patterns can be understood through the lens of bargaining theory: the absence of financial resources weakens a woman’s bargaining position within the household, limiting her capacity to exit or reshape unsafe relationship dynamics [13,14]. In line with gendered power theories, this economic dependence may also reinforce traditional gender hierarchies, allowing the male partner to exert greater coercive control [15]. Taken together, these findings highlight that structural disadvantage may contribute to heightened vulnerability to physical IPV during separation.

One alternative explanation for our findings is that women who are more economically independent may be in a better position to leave a relationship earlier, thereby reducing their cumulative exposure to physical IPV. In this scenario, the lower risk of experiencing physical IPV during separation among more educated and employed women could reflect not only a protective effect of economic independence, but also earlier exits from dysfunctional partnerships. While our panel data allows us to measure employment prior to separation to reduce concerns of reverse causality, we interpret the association with caution, as unobserved heterogeneity that affects both employment and IPV risk (such as family background, social support, or mental health) may still bias the results. Moreover, we cannot fully account for women’s prior exposure to physical IPV.

Our results highlight that insufficient integration of women into the labor market may indirectly influence levels of physical IPV during separation, particularly among less educated women. These findings underscore the importance of policies that support maternal employment. Expanding access to affordable, high-quality childcare can help mothers maintain continuous employment and reduce economic dependence. By enabling greater economic autonomy, such policies may help reduce vulnerability to physical IPV and support safer separation processes for mothers. Germany has introduced significant policies aimed at supporting women’s employment, such as the right to daycare for children aged one year and older (starting in 2013) and parental leave benefits (implemented in 2007). However, austerity measures threaten to reverse some of these positive developments. Additionally, labor shortages have led to reduced operating hours for daycare centers, making it increasingly difficult for women to engage in paid employment for more than (low) part-time hours. These developments may hinder women’s ability to achieve economic independence and could contribute to increased relationship conflict and heightened physical IPV risk around the time of separation.

While our study focuses on Germany, the observed patterns resonate with findings from other contexts. Overall, research from high-income countries similarly shows that women’s economic independence is associated with a reduced risk of IPV [10]. In contrast, evidence from low- and middle-income countries often points to more complex dynamics [11,18–20]. In some contexts, women’s increased economic resources are associated with lower IPV risk, while in others, they may initially provoke male backlash, particularly when gender norms are strongly patriarchal. This suggests that structural supports for women’s economic independence are relevant to IPV risk across diverse settings, but their effects may vary depending on broader cultural and institutional contexts.

This study generated novel evidence on the determinants of physical IPV during the separation process. However, this analysis is subject to several limitations. Empirical insights into the issue are highly sensitive to the operationalization of IPV in the data, including but not limited to the types of behavior surveyed, the time frame in which the surveyed behavior occurred, and which outcomes (such as incidence or severity) are analyzed. Moreover, the reporting of deviant behaviors and stigmatized experiences such as IPV is prone to social desirability bias. Victims may underreport victimization not only due to feelings of shame, fear, or self-blame, but also due to a lack of awareness or understanding of what constitutes violence [39–41]. Certain groups of women, such as those still in contact with an abusive partner or those experiencing strong emotional distress, may feel more constrained in answering questions regarding IPV. As a result, these groups might be underrepresented in the sample. Another limitation is that the analysis was limited to women who separated from their partners to allow us to focus on a well-defined moment in the life course when conflict and violence tends to escalate. The disadvantage of this approach is that we disregarded women who may have been unable to leave an abusive relationship. As a result, our results cannot be generalized, and are limited to women who are able to end dysfunctional and physically abusive relationships.
